# Two cases of subacute thyroiditis after different types of SARS-CoV-2 vaccination

**DOI:** 10.20945/2359-3997000000430

**Published:** 2022-01-14

**Authors:** Hayri Bostan, Ilknur Ozturk Unsal, Muhammed Kizilgul, Umran Gul, Muhammed Erkam Sencar, Bekir Ucan, Erman Cakal

**Affiliations:** 1 University of Health Sciences Diskapi Yildirim Beyazit Training and Research Hospital Department of Endocrinology and Metabolism Ankara Turkey Department of Endocrinology and Metabolism, University of Health Sciences, Diskapi Yildirim Beyazit Training and Research Hospital, Ankara, Turkey

## Abstract

Although the development of subacute thyroiditis (SAT) following viral infections is well-documented, the actual mechanism has not been clearly elucidated. The occurrence of SAT after vaccination has been reported in several case series and possible mechanisms such as molecular mimicry due to the exposure to viral proteins and/or abnormal reactogenicity by adjuvants have been implicated. We describe two cases who developed SAT three days after the messenger RNA vaccine against COVID-19 (Pfizer-BioNTech^®^) and six days after the inactivated COVID-19 vaccine (CoronaVac^®^). SAT diagnosis of these patients was delayed for more than two weeks. When the current cases were evaluated together with 1 Pfizer-BioNTech^®^ and 3 CoronaVac^®^ related cases reported previously, the patients were female aged between 30-42, except for the male patient we presented, and the complaints of the patients initiated within the first 2-7 days. While two Pfizer-BioNTech^®^ vaccine-related cases were severely symptomatic and thyrotoxic at presentation, there were cases with mild to moderate clinical manifestations in CoronaVac^®^ vaccine-related group. Physicians should be aware of SAT that may occur within a few days following the COVID-19 vaccination.

## INTRODUCTION

The outbreak of the severe acute respiratory syndrome coronavirus 2 (SARS-CoV-2) infection in late 2019 caused coronavirus disease 2019 (COVID-19), which soon became a global pandemic. Efforts to control this highly infectious disease are still ongoing 18 months later. The World Health Organization (WHO) has approved the emergency use of SARS-CoV-2 vaccines produced by different countries and as of June 2021, approximately 1.6 billion doses of vaccine have been administered worldwide ( 1 ).

Subacute thyroiditis (SAT) is a rare, self-limiting inflammatory disease of the thyroid gland ( 2 ). Viral pathogens are thought to play a role in the etiology, and SAT cases have been reported after COVID-19 infection ( 3 , 4 ). Vaccination-related SAT has been reported previously after influenza, human papilloma virus (HPV) and hepatitis B vaccines ( 5 - 8 ). The most important common feature of these vaccines is the use of aluminum-based adjuvants to increase immunogenicity. In recent years, it has been emphasized that adjuvants in vaccines may cause SAT and autoimmune thyroid diseases, as a part of ASIA (autoimmune/inflammatory syndrome induced by adjuvants) syndrome ( 8 , 9 ).

Currently, the data on SARS-CoV-2 vaccination-associated SAT is limited and the exact pathophysiological mechanism has not been elucidated as yet ( 10 - 12 ). In addition to possible ASIA syndrome, it has been speculated that the autoimmune diseases caused by current vaccines may be explained by the cross-reactivity that develops due to the presence of some peptide sequences that mimic mammalian cells in SARS-CoV-2 spike proteins ( 13 ).

The cases presented here are of two patients with SAT associated with different types of SARS-CoV-2 vaccines that have reached widespread use.

## CASE PRESENTATIONS

### Case 1

A 61-year-old male patient was referred to our clinic from the internal medicine department after overt hyperthyroidism was detected in laboratory tests. The patient, who has a history of diabetes mellitus and hypertension for 10 years, had COVID-19 infection in August 2020. There was no known history of allergy, autoimmune disease, or thyroid disease. The first dose of CoronaVac^®^ was administered 3 weeks before presentation on May 03, 2021. Six days after the vaccination, the patient started to feel swelling in the anterior region of the neck and pain radiating to the jaw. The patient then presented at the emergency room with the additional complaints of fever exceeding 38.5 °C, palpitations, fatigue, and excessive sweating. On the emergency admission, the COVID-19 RT-PCR (real-time reverse transcription polymerase chain reaction) test was negative. Amoxicillin/clavulanate was prescribed by the emergency physician to the patient who was found to have leukocytosis and elevated C-reactive protein levels. The patient reported that although his neck pain regressed relatively within days, the complaints of sweating, weakness and palpitations continued, and he had lost 5 kg in weight in the last 2 weeks.

**Figure 1:**
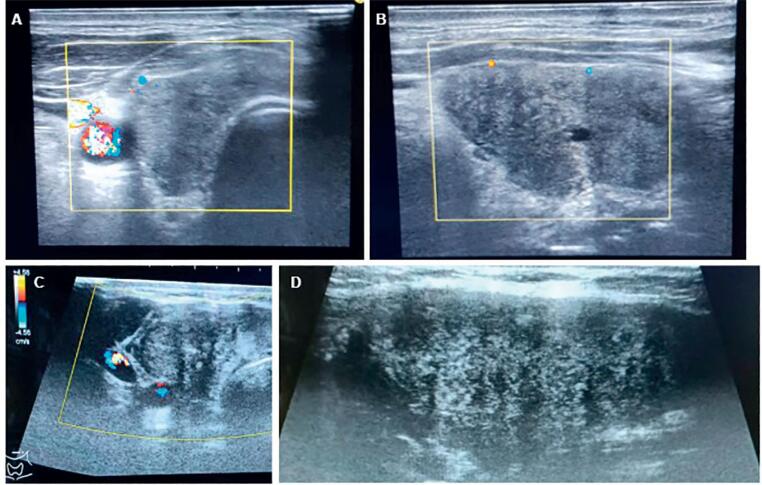
Thyroid ultrasonography (USG) images of the patients. Patchy hypoechoic areas with a lack of flow color on Doppler USG in the right lobe of thyroid gland in the transverse ( **A** ) and longitudinal planes in patient 1 ( **B** ), and the appearance of the same findings in the transverse ( **C** ) and longitudinal ( **D** ) planes in patient 2.

Physical examination revealed swelling and tenderness that limited the palpation, mostly on the right side of the thyroid gland at presentation. During the examination, temperature was 36.8 °C and heart rate was 98/min. Thyrotoxicosis and elevated ESR and CRP levels were observed in biochemical tests ( Table 1 ). Anti-thyroglobuline and anti-thyroid peroxidase antibodies were not detected. Thyroid US showed devascularized patchy hypoechoic areas in both lobes, more intensely in the right lobe, and diffuse enlargement in the thyroid gland ( Figure 1A-B ). The diagnosis of SAT was made based on the clinical presentation, biochemical tests and ultrasonographic findings of the patient in accordance with the 2016 American Thyroid Association guidelines ( 14 ). Ibuprofen 1,200 mg/day and propranolol 20 mg twice a day were started. Significant regression was observed in the clinical and laboratory findings during 2 week follow-up ( Table 2 ). Although subclinical hypothyroidism, which started in the first month after treatment, also continued at the follow-up in August 2021 [TSH: 5.34 mIU/L (reference range, 0.27-4.2), fT4:14.4 pmol/L (reference range, 11.97-21.88)], the patient did not experience any symptoms related to the hypothyroidism. In addition, the patient received the second dose of vaccine after normalization of free thyroid hormone levels and did not experience any symptoms in the post-vaccine period.

**Table 1:** Characteristics and biochemical test results of the patients

	Patient 1	Patient 2
Age (years)/Sex	61/M	32/F
Administered vaccine	CoronaVac^®^	Pfizer-BioNTech^®^
Post-vaccination symptom onset (days)	6	3
Time to diagnosis (days)	19	31
TSH (0.27-4.2 mIU/L)	0.02	<0.01
fT4 (11.97-21.88 pmol/L)	27.8	53.5
fT3 (3.08-6.78 pmol/L)	6.61	14.5
Anti-TPO (0-5.61 IU/mL)	0.51	2.32
Anti-Tg (0-4.11 IU/mL)	1.51	3.41
TRAb (<1.5 IU/L)	0.25	NA
WBC (3.57-11.01 10^3^/µL)	8.06	6.83
CRP (<5 mg/L)	28.6	24.09
ESR (0-20 mm/hour)	29	62

Anti-TPO: anti-thyroid peroxidase antibodies; Anti-Tg: anti-thyroglobuline antibodies; CRP: C-reactive protein; ESR: erythrocyte sedimentation rate; NA: not assessed; TSH: thyroid-stimulating hormone; fT4: free thyroxin; fT3: free tri-iodothyronine; TRAb: TSH receptor antibodies; WBC: white blood cells.

**Table 2:** Reported cases of subacute thyroiditis associated SARS-CoV-2 vaccination

No	Author	Age/Sex	Vaccine	Sypmtom onset (days)	Disease severity at diagnosis	Treatment	Laboratory test results			Reference values
							At diagnosis		On follow-up	
1	Iremli and cols. (10)	35/F	CoronaVac^®^ – 2nd dose	4	Mild-to-moderate	MP 16 mg/day + Propranolol			At 4^th^ week	
							TSH	0.47	2.27	0.38-5.33 mIU/L
							fT4	14.1	14.8	7.86-14.41 pmol/L
							fT3	6.15	5.3	3.8-6 pmol/L
							ESR	53	28	<20 mm/h
							CRP	100.5	13.1	<5 mg/L
2		34/F	CoronaVac^®^ – 1st dose	4	Moderate-to-severe	MP 16 mg/day + Propranolol	TSH	0.01	<0.015	
							fT4	5.2	25.85
							fT3	11.8	8.02
							ESR	19	16
							CRP	6	5.3
3		37/F	CoronaVac^®^ – 2nd dose	7	Mild	No treatment	TSH	0.9	0.018	
							fT4	13.8	26.1
							fT3	6.05	6.99
							ESR	25	44
							CRP	2.4	ND
4	Franquemont and Galvez (11)	42/F	Pfizer-BioNTech^®^ – 1st dose	5	Moderate-to-severe	Prednisone 40 mg/day + Propranolol			At 3^rd^ week	
							TSH	<0.01	<0.01	ND (mIU/L)
							fT4	58.9	41.2	ND (pmol/L)
							fT3	18.2	ND	ND (pmol/L)
							ESR	62	26	ND (mm/h)
5	Oyibo (12)	55/F	Vaxzevria^®^– 1st dose	21	Mild	Ibuprofen + Propranolol			At 6^th^ week	
							TSH	0.09	20.3	0.3-4.2 mIU/L
							fT4	25.2	4.7	12.0-22.0 pmol/L
							ESR	51	ND	0-18 mm/h
							CRP	87	ND	<5 mg/L
6	Current patient 1	61/M	CoronaVac^®^ – 1^st^ dose	6	Mild	Ibuprofen + Propranolol			At 2^nd^ week	
							TSH	0.02	0.1	0.27-4.2 mIU/L
							fT4	27.8	15.7	11.97-21.88 pmol/L
							fT3	6.61	4.51	3.08-6.78 pmol/L
							ESR	29	7	0-20 mm/h
							CRP	28.6	2.33	<5 mg/L
7	Current patient 2	32/F	Pfizer-BioNTech^®^ – 1st dose	3	Moderate-to-severe	MP 16 mg/day + Propranolol			At 3^rd^ week	
							TSH	<0.01	0.07
							fT4	53.5	12.5
							fT3	14.5	3.02
							ESR	62	4
							CRP	24.09	0.19

CRP: C-reactive protein; ESR: erythrocyte sedimentation rate; TSH: thyroid-stimulating hormone; fT4: free thyroxin; fT3: free tri-iodothyronine; MP: methylprednisolone; ND: not defined.

### Case 2

A 32-year-old female patient presented at our clinic with excessive sweating and severe pain located in the anterior neck region that limited the palpation of the thyroid gland. The patient had no known history of chronic disease and had been breastfeeding for 10 months. There was no known history of allergy, autoimmune disease, thyroid disease, or previous upper respiratory tract infection. The patient had received the first dose of Pfizer-BioNTech^®^ vaccine on April 28, 2021, 1 month before presentation. Three days after the vaccination, she started to feel complaints of neck pain, arthralgia, and fever exceeding 38 °C. On presentation at the internal medicine clinic, the COVID-19 RT-PCR test was found to be negative and paracetamol and antibiotic were prescribed. Despite the intermittent use of paracetamol, the neck pain did not regress and the complaints of palpitations and sweating increased, and finally, the patient lost 7 kg in 3 weeks.

On physical examination, there was tenderness and swelling in both thyroid lobes, more prominently in the right thyroid lobe with palpation. The other systemic examinations were unremarkable. There was significant thyrotoxicosis and elevated ESR and CRP levels in the laboratory tests ( Table 1 ). Thyroid US revealed diffuse swelling of both thyroid glands. There were patchy hypoechoic areas with a lack of flow color on Doppler US in both thyroid lobes, especially in the right lobe ( Figure 1C-D ). The diagnosis of SAT was confirmed and methylprednisolone 16 mg/day and propranolol 20 mg twice a day were prescribed. Three weeks after the initiation of steroid treatment, significant improvement was observed in clinical and laboratory examinations ( Table 2 ).

## DISCUSSION

The COVID-19 vaccination programs are continuing rapidly all over the world with the combination of different SARS-CoV-2 vaccine types. While the programs are sustained, side-effects that may develop after vaccination are also carefully monitored. Post-vaccination SAT is a rarely encountered entity ( 5 - 7 ). The first reports of SAT following SARS-CoV-2 vaccination were published in May 2021 ( 10 , 11 ). In this paper, the cases are described of 2 patients who developed SAT after CoronaVac^®^ and Pfizer-BioNTech^®^ vaccines, which are currently in use in many countries, and the cases are reviewed in the light of the literature.

SAT is a painful inflammatory disease of the thyroid gland. Although the etiology is not fully understood, viral pathogens are thought to be responsible ( 2 , 4 ). SAT cases have also been reported after SARS-CoV-2 virus infection ( 3 ). Angiotensin-converting enzyme 2 (ACE2) and transmembrane protease serine 2 (TMPRSS2), which are important for the entry of SARS-CoV-2 into the human cell, are highly expressed in the thyroid gland in both sexes ( 15 ). Rotondi and cols. detected ACE2 mRNA in thyroid follicular cells, and stated that this may be explanatory for SAT developing during or after the course of COVID-19 infection ( 16 ). Therefore, the direct virus effect seems to be also important in SARS-CoV-2-associated SAT cases. In addition, genetic predisposition is thought to be another factor in the etiology of SAT ( 17 , 18 ). Stasiak and cols. stressed that carrying some human leukocyte antigens (HLA) such as HLA-B*18:01 and DRB1*01 is an independent risk factor for SAT ( 18 ).

Pfizer-BioNTech^®^ is a nucleoside modified messenger RNA (modRNA) vaccine containing an RNA-lipid fragment encoding the spike glycoprotein of SARS-CoV-2, which was authorized for emergency use by the U.S. Food and Drug Administration (FDA) in December 2020 ( 19 ). Encapsulating RNA with lipid content facilitates transfection of mRNA ( 20 ). Polyethylene glycol (PEG) is used to stabilize the lipid content of the vaccine. Pfizer-BioNTech^®^ vaccination-associated SAT was reported in a 42-year-old female patient, apart from our case ( 11 ). In addition, Vera-Lastra and cols. reported 2 cases of Pfizer-BioNTech^®^ vaccination-associated Graves’ disease (GD) ( 21 ). In these reported cases, symptom onset is 2 to 5 days after vaccination ( 11 , 21 ). Despite the late diagnosis, both Pfizer-BioNTech^®^ vaccine-related SAT cases, including the case we described, were severely symptomatic and thyrotoxic at presentation ( Table 2 ).

Adjuvants are commonly used in vaccines to increase the immune response ( 22 ). Adjuvants also increase the amount of vaccine that can be produced by enabling the use of less viral antigen ( 9 ). However, in some predisposed individuals, adjuvants can also trigger autoimmune/inflammatory conditions. This phenomenon was first described as ASIA syndrome in 2011 ( 9 ). Although the Pfizer-BioNTech^®^ vaccine did not contain any known adjuvants, Vera-Lastra and cols. speculated that PEG could trigger immune reactions by acting as an adjuvant and cause ASIA syndrome ( 21 , 23 ). However, considering the SAT cases reported after Pfizer-BioNTech^®^ vaccine, the relatively different disease severity compared to CoronaVac^®^ related-cases indicates that different pathophysiological mechanisms may be effective. Moreover, a case of SAT associated with Vaxzevria^®^ (Adenovirus vectored COVID-19 vaccine, AstraZeneca^®^) without any adjuvant has also been reported recently ( Table 2 ) ( 12 ). It has been shown that the SARS-CoV-2 spike protein shares hexa and heptapeptides with mammalian proteomes, and it has been suggested to minimize these similar proteins in vaccines in order to minimize possible advers autoimmune conditions ( 13 , 23 ). Pfizer-BioNTech^®^ vaccine contains mRNA encoding SARS-CoV-2 spike protein and viral protein production reaches peak levels within 24-48 hours after vaccination, so humoral T cell-mediated immune response is triggered afterwards ( 24 ). In this immune-reactive environment, cross-reactivity that may occur due to molecular mimicry of SARS-CoV-2 spike proteins with thyroid peroxidase peptides seems to be a possible pathophysiological mechanism to trigger autoimmune/inflammatory thyroid disorders in predisposed individuals ( 13 , 23 ). Furthermore, the fact that the current case was in the postpartum period and therefore had increased immune reactivity may be another factor that facilitated the development of SAT ( 25 ).

CoronaVac^®^ (Sinovac Life Sciences, Beijing, China) is an inactivated COVID-19 vaccine created from African green monkey kidney cells (vero cells) inoculated with SARS-CoV-2 ( 26 ). Just as with inactivated seasonal flu vaccines, an adjuvant containing aluminum hydroxide is used in CoronaVac^®^ to increase immunogenicity. CoronaVac^®^ has been implemented in Turkey since January 2021, and the WHO validated the emergency use authorization for the vaccine as of June 2021 ( 27 ). To date, 3 more cases of CoronoVac^®^ vaccination-related SAT have been reported from Turkey, in addition to the current case ( 10 ). Symptoms developed between days 4 and 7 post-vaccination in all patients. The current case was the first report of a male patient with COVID-19 vaccination-associated SAT. SAT developed after the first dose of the vaccine and thyrotoxicosis was more pronounced in the current case, but steroid treatment was not considered because the clinical condition was mild and tended to improve. In 2 of the previously reported cases, SAT developed after the second dose of the vaccine, and the patients were euthyroid at the time of diagnosis, and one of these patients improved without any treatment ( 10 ) ( Table 2 ). In addition, fT4 levels were not elevated in any of these 3 reported patients at the time of diagnosis. The overlap of available data with previously reported cases of vaccine-associated (such as influenza, HBV, HPV) SAT and the use of aluminum-based adjuvants in these vaccines ( 5 - 7 ), including CoronaVac^®^, suggest that the possible pathophysiological mechanism is inappropriate reactogenicity in the thyroid gland, which develops due to the adjuvant, in other words ASIA syndrome ( 8 , 9 ).

Considering the cases reported after the COVID-19 vaccination ( 21 ), GD should be considered in the differential diagnosis in the approach to post-vaccine hyperthyroidism. In both current cases, the presence of typical ultrasonographic and clinical findings of SAT at presentation and rapid improvement of the free thyroid hormone levels without anti-thyroid treatment in a short period ruled out a possible diagnosis of GD.

It appears that COVID-19 vaccine-associated SAT occurs within the first week after vaccination. There are delays in the diagnosis of SAT due to low awareness of physicians and antibiotics are prescribed unnecessarily ( 28 , 29 ). In both cases presented in this paper, SAT could not be detected at the first examination and antibiotics were recommended to the patients, but case 2 did not use antibiotics. Even though these reported cases of SAT developed after vaccination, a rare indolent disease such as SAT should not discourage patients from being vaccinated against COVID-19. In cases that develop SAT after the first vaccination, as in current cases, the vaccination program should be sustained when clinical and laboratory improvement occurs.

In conclusion, during this period when COVID-19 vaccination continues rapidly, SAT should be considered in patients describing anterior neck pain after vaccination and unnecessary antibiotic prescriptions should be avoided. While most patients who develop SAT after COVID-19 vaccination are females aged 30-40 years, an elderly male is reported for the first time in this presentation. The pathophysiological mechanism of vaccine-associated SAT is still not elucidated. The clinical heterogeneity of these SAT cases reported after three different COVID-19 vaccines suggests that multiple mechanisms may be involved in vaccine-associated SAT. As suggested in previous studies, minimizing viral proteins shared with human proteins in vaccines seems to be a step that will increase the safety and tolerability of these vaccines ( 13 , 23 ). Furthermore, SAT are thought to be triggered in predisposed individuals. It seems important that future studies focus on elucidating the HLA alleles associated with post-vaccination SAT.
